# An Existence Theorem for Fractional *q*-Difference Inclusions with Nonlocal Substrip Type Boundary Conditions

**DOI:** 10.1155/2015/424306

**Published:** 2015-01-05

**Authors:** Ahmed Alsaedi, Sotiris K. Ntouyas, Bashir Ahmad

**Affiliations:** ^1^Department of Mathematics, Faculty of Science, King Abdulaziz University, P.O. Box 80203, Jeddah 21589, Saudi Arabia; ^2^Department of Mathematics, University of Ioannina, 451 10 Ioannina, Greece

## Abstract

By employing a nonlinear alternative for contractive maps, we investigate the existence of solutions for a boundary value problem of fractional *q*-difference inclusions with nonlocal substrip type boundary conditions. The main result is illustrated with the aid of an example.

## 1. Introduction

In this paper, we consider the following boundary value problem of fractional *q*-difference inclusions with nonlocal and substrip type boundary conditions:

(1) $$\begin{array}{c} {\;}^{c}D_{q}^{\upsilon }x\left(t\right)\in F\left(t,x\left(t\right)\right),\;t\in \left[0,1\right],\;\;1<\upsilon \leq 2,\;\;0<q<1,\\ x\left(0\right)=g\left(x\right),\;\;x\left(\omega \right)=b\overset{1}{\underset{\delta }{\int }}x\left(s\right)d_{q}s,\;0<\omega <\delta <1, \end{array}$$

where ^*c*^ *D*_*q*_^*υ*^ denotes the Caputo fractional *q*-derivative of order *υ*, *F* : [0,1] × *R* → *P*(*R*) is a multivalued map, *P*(*R*) is the family of all nonempty subsets of *R*, *g* : *C*([0,1], *R*) → *R* is a given continuous function, and *b* is a real constant. Here, we emphasize that the nonlocal conditions are regarded as more plausible than the standard initial conditions for the description of some physical phenomena. In (1), *g*(*x*) may be understood as *g*(*x*) = ∑_*j*=1_^*p*^ *α*_*j*_ *x*(*t*_*j*_), where *α*_*j*_, *j* = 1,…, *p*, are given constants and 0 < *t*_1_ < ⋯<*t*_*p*_ ≤ 1. For more details, we refer to the work by Byszewski and Lakshmikantham [1, 2].

Boundary value problems with integral boundary conditions constitute an important class of problems and arise in the mathematical modeling of various phenomena such as heat conduction, wave propagation, gravitation, chemical engineering, underground water flow, thermoelasticity, and plasma physics. They include two-point, three-point, multipoint, and nonlocal boundary value problems.

The topic of fractional differential equations has been of great interest for many researchers in view of its theoretical development and widespread applications in various fields of science and engineering such as control, porous media, electromagnetic, and other fields [3, 4]. For some recent results and applications, we refer the reader to a series of papers ([5–13]) and the references cited therein.

Fractional *q*-difference (*q*-fractional) equations are regarded as fractional analogue of *q*-difference equations. Motivated by recent interest in the study of fractional-order differential equations, the topic of *q*-fractional equations has attracted the attention of many researchers. The details of some recent work on the topic can be found in ([14–20]). For notions and basic concepts of *q*-fractional calculus, we refer to a recent text [21].

The present work is motivated by a recent paper of the authors [22], where the problem (1) was considered for a single valued case. To the best of our knowledge, this is the first paper dealing with fractional *q*-difference inclusions in the given framework. Moreover, the main result of our paper can be regarded as an improvement and extension of some known results; see, for instance, [18, 19].

The paper is organized as follows.  Section 2 contains some fundamental concepts of fractional *q*-calculus. In Section 3, we show the existence of solutions for the problem (1) by means of the nonlinear alternative for contractive mappings. Finally, an example illustrating the applicability of our result is presented.

## 2. Preliminaries

First of all, we recall the notations and terminology for *q*-fractional calculus [21, 23, 24].

For a real parameter *q* ∈ *R* ^+^∖{1}, a *q*-real number denoted by [*a*]_*q*_ is defined by

(2) $$\begin{array}{c} {\left[a\right]}_{q}=\frac{1-q^{a}}{1-q},\;a\in R. \end{array}$$

The *q*-analogue of the Pochhammer symbol (*q*-shifted factorial) is defined as

(3) $$\begin{array}{c} {\left(a;q\right)}_{0}=1,\;{\left(a;q\right)}_{k}=\overset{k-1}{\underset{i=0}{\prod }}\left(1-aq^{i}\right),\;k\in N\cup \left\{\infty \right\}. \end{array}$$

The *q*-analogue of the exponent (*x* − *y*)^*k*^ is

(4) x−y0=1, x−yk=∏j=0k−1x−yqj,0000000000000000000000k∈N, x,y∈R.

The *q*-gamma function Γ_*q*_(*y*) is defined as

(5) $$\begin{array}{c} \Gamma_{q}\left(y\right)=\frac{{\left(1-q\right)}^{\left(y-1\right)}}{{\left(1-q\right)}^{y-1}}, \end{array}$$

where *y* ∈ *R*∖{0, −1, −2,…}. Observe that Γ_*q*_(*y* + 1) = [*y*]_*q*_Γ_*q*_(*y*).

**Definition 1 (see [23]).**

Let *f* be a function defined on [0,1]. The fractional *q*-integral of the Riemann-Liouville type of order *β* ≥ 0 is (*I*_*q*_^0^ *f*)(*t*) = *f*(*t*) and

(6) Iqβft≔∫0tt−qsβ−1Γqβfsdqs=tβ1−qβ∑k=0∞qkqβ;qkq;qkftqk,000000000000000β>0, t∈0,1.

Observe that *β* = 1 in Definition 1 yields *q*-integral:

(7) $$\begin{array}{c} I_{q}f\left(t\right):=\overset{t}{\underset{0}{\int }}f\left(s\right)d_{q}s=t\left(1-q\right)\overset{\infty }{\underset{k=0}{\sum }}q^{k}f\left(tq^{k}\right). \end{array}$$

For more details on *q*-integrals and fractional *q*-integrals, see Sections 1.3 and 4.2, respectively, in [21].

**Remark 2 .**

The *q*-fractional integration possesses the semigroup property ([21, Proposition 4.3]):

(8) $$\begin{array}{c} I_{q}^{\gamma }I_{q}^{\beta }f\left(t\right)=I_{q}^{\beta +\gamma }f\left(t\right);\;\gamma ,\beta \in R^{+}. \end{array}$$

Further, it has been shown in Lemma 6 of [24] that

(9) Iqβxσ=Γqσ+1Γqβ+σ+1xβ+σ,0<x<a, β∈R+, σ∈−1,∞.

Before giving the definition of fractional *q*-derivative, we recall the concept of *q*-derivative.

We know that the *q*-derivative of a function *f*(*t*) is defined as

(10) $$\begin{array}{c} \left(D_{q}f\right)\left(t\right)=\frac{f\left(t\right)-f\left(qt\right)}{t-qt},\;t\neq 0,\\ \left(D_{q}f\right)\left(0\right)=\underset{t\to 0}{\lim}\left(D_{q}f\right)\left(t\right). \end{array}$$

Furthermore,

(11) $$\begin{array}{c} D_{q}^{0}f=f,\;D_{q}^{n}f=D_{q}\left(D_{q}^{n-1}f\right),\;n=1,2,3,\ldots . \end{array}$$

**Definition 3 (see [21]).**

The Caputo fractional *q*-derivative of order *β* > 0 is defined by

(12) $$\begin{array}{c} {\;}^{c}D_{q}^{\beta }f\left(t\right)=I_{q}^{\left\lceil \beta \right\rceil -\beta }D_{q}^{\left\lceil \beta \right\rceil }f\left(t\right), \end{array}$$

where ⌈*β*⌉ is the smallest integer greater than or equal to *β*.

Next, we recall some properties involving Riemann-Liouville *q*-fractional integral and Caputo fractional *q*-derivative ([21, Theorem 5.2]):

(13) Iqβ  cDqβft=ft−∑k=0β−1tkΓqk+1Dqkf0+,000000000000000000000∀t∈0,a, β>0;  cDqβ  Iqβf(t)=f(t), ∀t∈(0,a],  β>0.

**Lemma 4 (see [22]).**

Let *y* ∈ *C*([0,1], *R*). Then, the following problem

(14) $$\begin{array}{c} {\;}^{c}D_{q}^{\upsilon }x\left(t\right)=y\left(t\right),\;1<\upsilon \leq 2,\\ x\left(0\right)=y_{0},\;x\left(\omega \right)=b\overset{1}{\underset{\delta }{\int }}x\left(s\right)d_{q}s,\;y_{0}\in R,\;\;t\in \left[0,1\right], \end{array}$$

is equivalent to an integral equation:

(15) xt=∫0tt−qs(υ−1)Γq(υ)y(s)dqs +tϑb∫δ1∫0ss−quυ−1Γqυyudqudqs    − ∫0ωω−qsυ−1Γqυysdqs +y01+tϑb1−δ−1,

where

(16) $$\begin{array}{c} \vartheta =\omega -\frac{b\left(1-\delta^{2}\right)}{1+q}\neq 0. \end{array}$$

## 3. Existence Results

First of all, we outline some basic definitions and results for multivalued maps [25, 26].

For a normed space (*X*, ‖·‖), let *P*_*cl*_(*X*) = {*Y* ∈ *P*(*X*) : *Y* is closed}, *P*_*b*_(*X*) = {*Y* ∈ *P*(*X*) : *Y* is bounded}, *P*_*cp*_(*X*) = {*Y* ∈ *P*(*X*) : *Y* is compact}, and *P*_*cp*,*c*_(*X*) = {*Y* ∈ *P*(*X*) : *Y* is compact and convex}. A multivalued map *G* : *X* → *P*(*X*)

(i) is convex (closed) valued if *G*(*x*) is convex (closed) for all *x* ∈ *X*;
(ii) is bounded on bounded sets if *G*(*B*) = ∪_*x*∈*B*_ *G*(*x*) is bounded in *X* for all *B* ∈ *P*_*b*_(*X*) (i.e., sup⁡_*x*∈*B*_{sup⁡{|*y* | :*y* ∈ *G*(*x*)}} < *∞*);
(iii) is called upper semicontinuous (u.s.c.) on *X* if, for each *x*_0_ ∈ *X*, the set *G*(*x*_0_) is a nonempty closed subset of *X* and if, for each open set *N* of *X* containing *G*(*x*_0_), there exists an open neighborhood *N*_0_ of *x*_0_ such that *G*(*N*_0_)⊆*N*;
(iv) is said to be completely continuous if *G*(*B*) is relatively compact for every *B* ∈ *P*_*b*_(*X*);
(v) is said to be measurable if, for every *y* ∈ *R*, the function
  (17) $$\begin{array}{c} t⟼d\left(y,G\left(t\right)\right)=\inf\left\{\left|y-z\right|:z\in G\left(t\right)\right\} \end{array}$$
  is measurable;
(vi) has a fixed point if there is *x* ∈ *X* such that *x* ∈ *G*(*x*). The fixed point set of the multivalued operator *G* will be denoted by Fix⁡*G*.

For each *x* ∈ *C*([0,1], *R*), define the set of selections of *F* by

(18) SF,x≔v∈L10,1,R:vt∈Ft,xt for  a.e.  t∈0,1.

**Definition 5 .**

A multivalued map *F* : [0,1] × *R* → *P*(*R*) is said to be a Carathéodory function if

- *t* ↦ *F*(*t*, *x*) is measurable for each *x* ∈ *R*;
- *x* ↦ *F*(*t*, *x*) is upper semicontinuous for almost all *t* ∈ [0,1].

Further, a Carathéodory function *F* is called *L* ^1^-Carathéodory if

(iii) for each *α* > 0, there exists *φ*_*α*_ ∈ *L* ^1^([0,1], *R* ^+^) such that
  (19) $$\begin{array}{c} \left\|F\left(t,x\right)\right\|=\sup\left\{\left|v\right|:v\in F\left(t,x\right)\right\}\leq \varphi_{\alpha }\left(t\right) \end{array}$$
 for all ‖*x*‖ ≤ *α* and for a.e. *t* ∈ [0,1].

We define the graph of *G* to be the set *Gr*(*G*) = {(*x*, *y*) ∈ *X* × *Y*,  *y* ∈ *G*(*x*)} and recall two results for closed graphs and upper-semicontinuity.

**Lemma 6 (see [25, Proposition 1.2]).**

If *G* : *X* → *P*_*cl*_(*Y*) is u.s.c., then *Gr*(*G*) is a closed subset of *X* × *Y*, that is, for every sequence {*x*_*n*_}_*n*∈*N*_ ⊂ *X* and {*y*_*n*_}_*n*∈*N*_ ⊂ *Y*, if *n* → *∞*, *x*_*n*_ → *x*_∗_, *y*_*n*_ → *y*_∗_, and *y*_*n*_ ∈ *G*(*x*_*n*_), then *y*_∗_ ∈ *G*(*x*_∗_). Conversely, if *G* is completely continuous and has a closed graph, then it is upper semicontinuous.

**Lemma 7 (see [27]).**

Let *X* be a separable Banach space. Let *F* : [0,1] × *X* → *P*_*cp*,*c*_(*X*) be an *L* ^1^-Carathéodory function. Then, the operator

(20) Θ∘SF:C0,1,X⟶Pcp,cC0,1,X,Θ∘SF:x⟼Θ∘SFx=ΘSF,x,y

is a closed graph operator in *C*([0,1], *X*) × *C*([0,1], *X*).

**Definition 8 .**

A function *x* ∈ *AC* ^1^([0,1], *R*) is called a solution of problem (1) if there exists a function *f* ∈ *L* ^1^([0,1], *R*) with *f*(*t*) ∈ *F*(*t*, *x*(*t*)), a.e. on [0,1] such that *D*_*q*_^*υ*^ *x*(*t*) = *f*(*t*), a.e. on [0,1] and *x*(0) = *g*(*x*) and *x*(*ω*) = *b*∫_*δ*_^1^ *x*(*s*)*d*_*q*_ *s*.

To prove our main result in this section we will use the following form of the nonlinear alternative for contractive maps [28, Corollary 3.8].

**Theorem 9 .**

Let *X* be a Banach space and let *D* be a bounded neighborhood of 0 ∈ *X*. Let *χ*_1_ : *X* → *P*_*cp*,*c*_(*X*) and $\chi_{2}:\overset{-}{D}\to P_{cp,c}(X)$ be two multivalued operators such that

- *χ*_1_ is contraction,
- *χ*_2_ is u.s.c and compact.

Then, if *χ* = *χ*_1_ + *χ*_2_, either

- *χ* has a fixed point in $\overset{-}{D}$ or
- there is a point *u* ∈ ∂*D* and *λ* ∈ (0,1) with *u* ∈ *λχ*(*u*).

**Theorem 10 .**

Assume that

(H_1_) *F* : [0,1] × *R* → *P*_*cp*,*c*_(*R*) is *L* ^1^-Carathéodory multivalued map;
(H_2_) there exists a continuous nondecreasing function *ψ* : [0, *∞*)→(0, *∞*) and a function *p* ∈ *C*([0,1], *R* ^+^) such that
  (21) F(t,x)P≔sup⁡y:y∈Ft,x≤ptψx00000000000000000for  each  t,x∈0,1×R;
(H_3_) *g* : *C*([0,1], *R*) → *R* is a continuous function satisfying the condition
  (22) $$\begin{array}{c} |g\left(u\right)-g\left(v\right)|\leq l\left\|u-v\right\|\;\forall u,v\in C\left(\left[0,1\right],R\right),\;\;l>0; \end{array}$$
(H_4_) there exists a number *M* > 0 such that
  (23) $$\begin{array}{c} \frac{\left(1-lk_{0}\right)M}{||p||\psi \left(M\right)\mu_{0}}>1, \end{array}$$
 with *lk*_0_ < 1, where
  (24) $$\begin{array}{c} \mu_{0}≔\frac{1}{\Gamma_{q}\left(\upsilon +1\right)}+\frac{1}{\left|\vartheta \right|}\left\{\frac{\left|b\right|\left(1-\delta^{\upsilon +1}\right)}{\Gamma_{q}\left(\upsilon +2\right)}+\frac{\omega^{\upsilon }}{\Gamma_{q}\left(\upsilon +1\right)}\right\},\\ k_{0}:=1+\frac{1}{\left|\vartheta \right|}\left|b\left(1-\delta \right)-1\right|. \end{array}$$

Then, the problem (1) has at least one solution on [0,1].

**Proof**

To transform the problem (1) into a fixed point problem, we define an operator *F* : *C*([0,1], *R*) → *P*(*C*([0,1], *R*)) as

(25) Fx=∫0tt−qs(υ−1)Γq(υ)h∈C([0,1],R):ht  =∫0tt−qs(υ−1)Γq(υ)f(s)dqs   +tϑb∫δ1∫0ss−quυ−1Γqυfudqudqs      − ∫0ωω−qsυ−1Γqυfsdqs   +1+tϑb1−δ−1gx,  t∈0,1, f∈SF,x∫0tt−qsυ−1Γqυ.

Next, we introduce two operators *F*_1_ : *C*([0,1], *R*) → *C*([0,1], *R*) and *F*_2_ : *C*([0,1], *R*)→*P*(*C*([0,1], *R*)) as follows:

(26) F1x(t)=1+tϑb1−δ−1g(x), t∈[0,1],F2x=∫0tt−qs(υ−1)Γq(υ)h∈C([0,1],R):h(t)  =∫0tt−qs(υ−1)Γq(υ)f(s)dqs   +tϑb∫δ1∫0ss−quυ−1Γqυfudqudqs      − ∫0ωω−qs(υ−1)Γq(υ)f(s)dqs,  t∈0,1, f∈SF,x∫0tt−qsυ−1Γqυ.

Observe that *F* = *F*_1_ + *F*_2_. We will show that the operators *F*_1_ and *F*_2_ satisfy all the conditions of Theorem 9 on [0,1]. For the sake of clarity, we split the proof into a number of steps and claims.

*Step 1. F*_1_ is a contraction on *C*([0,1], *R*). This is a consequence of (H_3_). Indeed, we have

(27) F1xt−F1yt=1+tϑb1−δ−1gx−gy≤1+1ϑb1−δ−1lx−y≤k0lx−y.

Taking supremum over *t* ∈ [0,1], we have

(28) $$\begin{array}{c} \left\|F_{1}x-F_{1}y\right\|\leq k_{0}l\left\|x-y\right\|,\;lk_{0}<1. \end{array}$$

*Step 2. F*_2_ is compact, convex valued, and completely continuous. This will be established in several claims.

*Claim 1. F*_2_ maps bounded sets into bounded sets in *C*([0,1], *R*). For that, let *B*_*ρ*_ = {*x* ∈ *C*([0,1], *R*) : ‖*x*‖ ≤ *ρ*} be a bounded set in *C*([0,1], *R*). Then, for each *h* ∈ *F*_2_(*x*), *x* ∈ *B*_*ρ*_, we have

(29) ht≤∫0tt−qsυ−1Γqυfsdqs +1|ϑ|b∫δ1∫0ss−quυ−1Γqυfudqudqs    + ∫0ωω−qs(υ−1)Γq(υ)fsdqs≤pψρ1Γqυ+1+1ϑb1−δυ+1Γqυ+2             + ωυΓqυ+11Γqυ+1+1ϑb1−δυ+1Γqυ+2≤pψ(ρ)μ0,

and consequently, for each *h* ∈ *F*_2_(*B*_*ρ*_), we have

(30) $$\begin{array}{c} \left\|h\right\|\leq \left\|p\right\|\psi \left(\rho \right)\mu_{0}. \end{array}$$

*Claim 2. F*_2_ maps bounded sets into equicontinuous sets. As before, let *B*_*ρ*_ be a bounded set and let *h* ∈ *F*_2_(*x*) for *x* ∈ *B*_*ρ*_. Let *t*_1_, *t*_2_ ∈ [0,1] with *t*_1_ < *t*_2_ and *x* ∈ *B*_*ρ*_. Then, for each *h* ∈ *F*_2_(*x*), we obtain

(31) F1xt2−F1xt1≤1Γqυ∫0t1t2−qsυ−1−t1−qsυ−1fsdqs +1Γqυ∫t1t2t2−qsυ−1fsdqs +t2−t1ϑb∫δ1∫0ss−quυ−1Γυfudqudqs      + ∫0ωω−quυ−1Γqυfsdqs≤pψρΓqυ∫0t1t2−qsυ−1−t1−qsυ−1dqs +pψρΓqυ∫t1t2t2−qs(υ−1)dqs +pψρt2−t1ϑb∫δ1∫0ss−quυ−1Γqυdqudqs          + ∫0ωω−qs(υ−1)Γq(υ)dqs,

which is independent of *x* and tends to zero as *t*_2_ − *t*_1_ → 0. Therefore, it follows by the Arzelá-Ascoli theorem that *F*_2_ : *C*([0,1], *R*) → *P*(*C*[0,1], *R*) is completely continuous.

*Claim 3. F*_2_ has a closed graph. Let *x*_*n*_ → *x*_∗_, *h*_*n*_ ∈ *F*_2_(*x*_*n*_), and *h*_*n*_ → *h*_∗_. Then, we need to show that *h*_∗_ ∈ *F*_2_(*x*_∗_). Associated with *h*_*n*_ ∈ *F*_2_(*x*_*n*_), there exists *f*_*n*_ ∈ *S*_*F*,*x*_*n*__ such that, for each *t* ∈ [0,1],

(32) hnt=∫0tt−qs(υ−1)Γq(υ)fn(s)dqs +tϑb∫δ1∫0ss−quυ−1Γqυfnudqudqs    − ∫0ωω−qs(υ−1)Γq(υ)fn(s)dqs.

Then, we have to show that there exists *f*_∗_ ∈ *S*_*F*,*x*_∗__ such that, for each *t* ∈ [0,1],

(33) h∗t=∫0tt−qs(υ−1)Γq(υ)f∗(s)dqs +tϑb∫δ1∫0ss−quυ−1Γqυf∗udqudqs    − ∫0ωω−qs(υ−1)Γq(υ)f∗(s)dqs.

Let us consider the continuous linear operator Θ : *L* ^1^([0,1], *R*) → *C*([0,1], *R*) defined by

(34) f⟼Θft=∫0tt−qsυ−1Γqυfsdqs +tϑb∫δ1∫0ss−quυ−1Γqυfudqudqs    − ∫0ωω−qs(υ−1)Γq(υ)f(s)dqs.

Observe that

(35) hnt−h∗t=∫0tt−qsυ−1Γqυfns−f∗sdqs  +tϑb∫δ1∫0ss−quυ−1Γqυfnu−f∗udqudqs    − ∫0ωω−qsυ−1Γqυfns−f∗sdqs⟶0,

as *n* → *∞*. Thus, it follows by Lemma 7 that Θ∘*S*_*F*_ is a closed graph operator. Further, we have *h*_*n*_(*t*) ∈ Θ(*S*_*F*,*x*_*n*__). Since *x*_*n*_ → *x*_∗_, therefore, we have

(36) h∗t=∫0tt−qs(υ−1)Γq(υ)f∗(s)dqs +tϑb∫δ1∫0ss−quυ−1Γqυf∗udqudqs    − ∫0ωω−qs(υ−1)Γq(υ)f∗(s)dqs,

for some *f*_∗_ ∈ *S*_*F*,*x*_∗__. Hence, *F*_2_ has a closed graph (and therefore has closed values). In consequence, the operator *F*_2_ is compact valued.

Thus, the operators *F*_1_ and *F*_2_ satisfy hypotheses of Theorem 9 and hence, by its application, it follows that either condition (i) or condition (ii) holds. We show that the conclusion (ii) is not possible. If *x* ∈ *λF*_1_(*x*) + *λF*_2_(*x*) for *λ* ∈ (0,1), then there exists *f* ∈ *S*_*F*,*x*_ such that *x* = *λF*(*x*), that is,

(37) xt=λ∫0tt−qs(υ−1)Γq(υ)f(s)dqs +λtϑb∫δ1∫0ss−quυ−1Γqυfudqudqs    − ∫0ωω−qs(υ−1)Γq(υ)f(s)dqs +λ1+tϑb1−δ−1gx; t∈[0,1].

In consequence, we have

(38) xt≤∫0tt−qs  υ−1Γqυfsdqs +1ϑb∫δ1∫0ss−quυ−1Γqυfudqudqs    + ∫0ωω−qs(υ−1)Γq(υ)fsdqs +1+1ϑb1−δ−1lx≤pψ(x)+1ϑb1−δυ+1Γqυ+2+ωυΓqυ+11Γqυ+1       + 1ϑb1−δυ+1Γqυ+2+ωυΓqυ+1 +1+1ϑb1−δ−1lx≤pψ(x)μ0+k0lx.

If condition (ii) of Theorem 9 holds, then there exist *λ* ∈ (0,1) and *x* ∈ ∂*B*_*M*_ with *x* = *λF*(*x*), where *B*_*M*_ = {*x* ∈ *C*([0,1], *R*) : ‖*x*‖ ≤ *M*}. Then, *x* is a solution of *x* = *λF*(*x*) with ‖*x*‖ = *M*. Now, by the last inequality, we get

(39) $$\begin{array}{c} \frac{\left(1-lk_{0}\right)M}{\left\|p\right\|\psi \left(M\right)\mu_{0}}\leq 1, \end{array}$$

which contradicts (23). Hence, *F* has a fixed point on [0,1] by Theorem 9, and consequently the problem (1) has a solution. This completes the proof.

**Example 11 .**

Consider the following *q*-fractional boundary value problem:

(40) $$\begin{array}{c} {\;}^{c}D_{q}^{3/2}x\left(t\right)\in F\left(t,x\left(t\right)\right),\;t\in \left[0,1\right],\\ x\left(0\right)=\frac{1}{2}+\frac{1}{5}\tan^{-1}\left(x\left(\frac{1}{4}\right)\right),\;\;x\left(\frac{1}{4}\right)=\frac{1}{5}\overset{1}{\underset{3/4}{\int }}x\left(s\right)d_{q}s. \end{array}$$

Here, *υ* = 3/2, *q* = 1/2, *b* = 1/5, *ω* = 1/4, *δ* = 3/4, *l* = 1/15, and

(41) $$\begin{array}{c} F\left(t,x\right)=\left[\frac{3}{{\left(3+t\right)}^{2}}\frac{\left|x\right|}{1+\left|x\right|}+\frac{\cos x}{{\left(4+t\right)}^{2}}+\frac{1}{2},-\frac{1}{10}\right]. \end{array}$$

Obviously,

(42) sup⁡u:u∈Ft,x≤33+t2+14+t2+12=ptψx.

With the given values, it is found that *ϑ* = 0.191667, *μ*_0_ ≈ 1.3990, *k*_0_ = 3.9564, ||*p*|| = 43/48, *ψ*(||*x*||) = 1, and *M* > 1.7026 by (H_3_). Thus, all the conditions of Theorem 10 hold. In consequence, the conclusion of Theorem 10 applies to the boundary value problem (40).
